# Bringing signaling complexity into focus

**DOI:** 10.7554/eLife.106519

**Published:** 2025-04-01

**Authors:** Sarah Y Coomson, Salil A Lachke

**Affiliations:** 1 Department of Biological Sciences, University of Delaware Newark United States; 2 Department of Biological Sciences and Center for Bioinformatics and Computational Biology, University of Delaware Newark United States

**Keywords:** lens, signaling, development, fibroblast growth factor signalling, eye, vision, Mouse

## Abstract

A study in mice reveals key interactions between proteins involved in fibroblast growth factor signaling and how they contribute to distinct stages of eye lens development.

**Related research article** Wang Q, Li H, Mao Y, Garg A, Park ES, Wu Y, Chow A, Peregrin J, Zhang X. 2024. Shc1 cooperates with Frs2 and Shp2 to recruit Grb2 in FGF-induced lens development. *eLife* **13**:RP103615. doi: 10.7554/eLife.103615.

Ask yourself, what is necessary for you to read this article? And how are you able to discern between individual words or letters? Evolution has solved these challenges by developing a complex organ, the eye, that is similar in many ways to a camera. It has a lens that focuses light onto a light-sensitive tissue, the retina, which captures this information and relays it to the brain. For the lens to be effective, it needs to be transparent, and complex genetic pathways have emerged to ensure that this happens during development. In vertebrates, the majority of the cells in the lens are fiber cells.

Although the need for cross-talk between the lens and the retina during lens development was first noticed in 1901 (Spemann, 1901), the details of the impact of this cross-talk on the differentiation of lens cells did not become clear until the early 1960s. Reversing the orientation of a chick embryonic lens so that its anterior region faced the retina (rather than looking outwards) led to a dramatic cellular reorganization: the lens epithelium facing the retina differentiated into fiber cells, suggesting that a key signal from the retina drives this differentiation of lens cells (Coulombre and Coulombre, 1963). This signal was later revealed to be a protein called fibroblast growth factor (or FGF for short; Chamberlain and McAvoy, 1987), which, depending on its dosage, can induce epithelial cell proliferation or their differentiation into fiber cells in the lens (McAvoy and Chamberlain, 1989).

These findings ignited studies that revealed there are four FGF receptors, which interact with FGF protein ligands to drive lens development (Garcia et al., 2011; Lovicu and Overbeek, 1998; Zhao et al., 2008). FGF signaling was generally considered to operate as a linear pathway in the lens, where activating the FGF receptors triggers phosphorylation of an adaptor protein known as Frs2, which then recruits Shp2 and Grb2 proteins. This activates a signaling cascade known as Ras-MAPK, which drives cell proliferation, survival, and differentiation (Li et al., 2014; Madakashira et al., 2012). However, the full complexity of this pathway, including how it controls specific developmental events, remained unclear. Now, in eLife, Xin Zhang and colleagues at Columbia University – including Qian Wang, Hongge Li and Yingyu Mao as joint first authors – report new insights into FGF-induced lens development (Wang et al., 2025).

Using the power of mouse genetics, the team deleted or altered receptors and adaptor proteins involved in the FGF signaling pathway to build up a picture of how each protein impacts lens development. Deleting all four FGF receptors severely compromised early events in lens formation (Figure 1A). However, if only two receptors (1 and 2) were deleted, the outcome was different; the remaining FGF receptors were sufficient to induce lens development, but certain aspects of the development were defective (Figure 1B). These defects could be partially rescued by activating a Ras pathway (Kras), suggesting that the Ras-MAPK cascade is a key downstream mediator of FGF signaling in the lens (Figure 1C).

**Figure 1.**
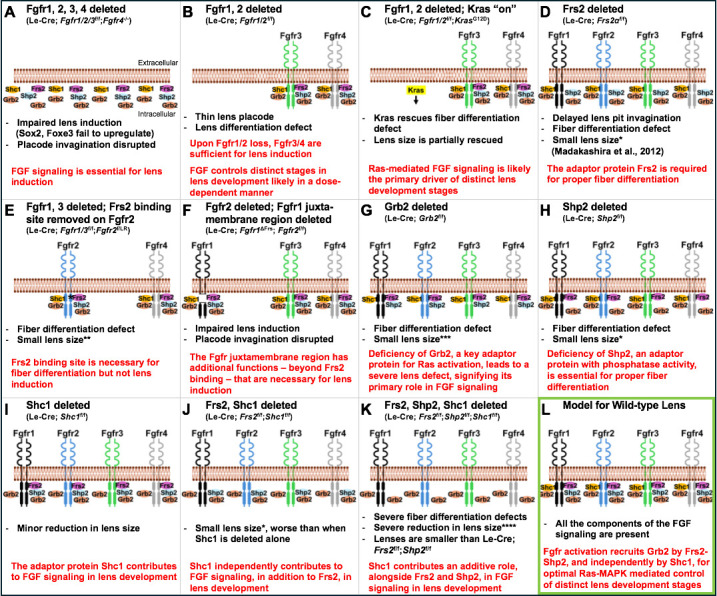
Genetic experiments in mice reveal the individual roles of key components of the FGF signaling pathway in lens development. (**A**) Deleting the genes for all four fibroblast growth factor receptors – Fgfr1, Fgfr2, Fgfr3 and Fgfr4 – impairs lens induction, prevents upregulation of key transcription factors known as Sox2 and Foxe3 and disrupts lens placode invagination. (**B**) When the genes for Fgfr1 and 2 are deleted, Fgfr3 (green) and Fgfr4 (grey) are sufficient to induce lens development, but there are defects in fiber cell differentiation. (**C**) Activating Kras signaling (highlighted in yellow) in the lenses of mice without the genes for Fgfr1 and 2 rescues the lens differentiation and lens size defects, likely by restoring downstream signaling events. (**D**) Deleting the gene for the adaptor protein Frs2 (pink) in the lens leads to fiber cell differentiation defects and reduced lens size. (**E**) Introducing a mutant version of Fgfr2 (blue) with a point mutation (denoted by asterisk) in the Frs2 binding site close to the plasma membrane in mice lacking Fgfr1–3 leads to fiber cell differentiation defects but not lens induction defects. (**F**) Deleting a larger juxtamembrane region (gap) in Fgfr1 (black) in mice lacking normal Fgfr1 and 2 results in severe lens defects, similar to those observed when all four receptors are lost. (**G**) Deleting the gene for the adaptor protein Grb2 (orange) leads to a severe fiber cell differentiation defect along with a major reduction in lens size. (**H**) Deleting the gene for the adaptor protein Shp2 (light blue) leads to lens fiber differentiation defects and a moderate reduction in lens size. (**I**) Deleting the gene for Shc1 (yellow) in the lens results in mild lens defects including minor reductions in lens size. (**J**) Deleting both Frs2 and Shc1 in the lens causes a reduction in lens size, which is more severe when compared to lenses deficient in Shc1 alone (as in (**I**)). (**K**) The absence of three adaptor proteins, Frs2, Shp2 and Shc1, results in severe lens fiber differentiation defects and grossly reduced lens size, dramatically smaller when compared to lenses from single gene or double gene deletions. (**L**) Together, these experiments support a model of FGF signaling in normal lens development (outlined in green), in which activation of FGF receptors leads to phosphorylation of Frs2. Frs2 then recruits Shp2, which subsequently recruits Grb2 and activates Ras-MAPK signaling, while Shc1 recruits Grb2 to the juxtamembrane region of the receptor, independently of Frs2. In the panel text, the number of * denotes the severity of the phenotype. FGF ligands, as well as phosphorylation events on either receptors or adaptors, are not shown. The extracellular and intracellular orientation retained in all panels is shown in (**A**). The genetic details of the mouse models corresponding to each panel are shown in brackets below the panel title.

Wang et al. next focused on interactions between Frs2 – an adaptor protein with a well-established role in FGF signaling (Madakashira et al., 2012) – and the FGF receptors. Experiments revealed that Frs2 must bind to a specific intracellular region of an FGF receptor to drive fiber cell differentiation, but this binding is not required for early lens development, suggesting that Frs2 has a critical role at later stages of the process (Figure 1D and E). Other experiments revealed a large portion of the receptor found close to the cell membrane is vital for lens induction and fiber differentiation, suggesting that factors (other than Frs2 binding) that interact with this receptor region are pivotal for early lens development (Figure 1F).

Focusing on Grb2 – a protein that is essential for activating Ras-MAPK signaling – Wang et al. showed that deleting the gene for Grb2 leads to defects in fiber cell differentiation but not early lens development (Figure 1G). While Grb2 binds to Frs2, mutating the Grb2-binding site in Frs2 did not cause lens defects. Further, deleting the adaptor protein Shp2, or introducing mutations that compromised its Grb2-binding and its ability to remove phosphates on other proteins, caused only mild lens defects (Figure 1H). This suggests that other adaptor proteins should be examined to identify their potential roles in lens development.

Finally, experiments focused on the adaptor protein Shc1 showed that it can function without binding to Frs2 but requires the larger protein region of the FGF receptor deleted in previous experiments. Furthermore, experiments deleting Shc1 alone, and then with other adaptor proteins (Frs2 and Shp2), revealed that defects became progressively more severe, supporting the idea that Shc1 has both an independent and additive role in FGF signaling in the lens (Figure 1I–K).

This tour de force study highlights how a complex network of adaptor proteins and compensatory mechanisms interact dynamically to precisely control key stages in lens development (Figure 1L). The findings also spark further questions: Does Shc1 (and/or other proteins) directly bind to the specific FGF reception region studied by Wang et al.? Are there additional components/adaptors that contribute to lens development? Understanding the complexities of a pathway as critical as FGF signaling is necessary for future strategies to treating lens disorders. Finally, as FGF signaling is central to numerous developmental processes, these findings in the lens highlight the potential for discovering new signaling relationships in other cells and tissues.
